# A primary care level algorithm for identifying HIV-infected adolescents in populations at high risk through mother-to-child transmission

**DOI:** 10.1111/j.1365-3156.2010.02708.x

**Published:** 2011-03

**Authors:** Rashida A Ferrand, Helen A Weiss, Kusum Nathoo, Chiratidzo E Ndhlovu, Stanley Mungofa, Shungu Munyati, Tsitsi Bandason, Diana M Gibb, Elizabeth L Corbett

**Affiliations:** 1 Department of Infectious and Tropical Diseases, London School of Hygiene and Tropical MedicineLondon, UK; 2 Biomedical Research and Training InstituteHarare, Zimbabwe; 3 Department of Epidemiology and Population Health, London School of Hygiene and Tropical MedicineUK; 4 Department of Paediatrics, University of ZimbabweHarare, Zimbabwe; 5 Department of Medicine, University of ZimbabweHarare, Zimbabwe; 6 Medical Research Council Clinical Trials UnitLondon, UK

**Keywords:** HIV, Primary Health Care, adolescent, mother-to-child transmission

## Abstract

**Objective:**

To present an algorithm for primary-care health workers for identifying HIV-infected adolescents in populations at high risk through mother-to-child transmission.

**Methods:**

Five hundred and six adolescent (10–18 years) attendees to two primary care clinics in Harare, Zimbabwe, were recruited. A randomly extracted ‘training’ data set (*n* = 251) was used to generate an algorithm using variables identified as associated with HIV through multivariable logistic regression. Performance characteristics of the algorithm were evaluated in the remaining (‘test’) records (*n* = 255) at different HIV prevalence rates.

**Results:**

HIV prevalence was 17%, and infection was independently associated with client-reported orphanhood, past hospitalization, skin problems, presenting with sexually transmitted infection and poor functional ability. Classifying adolescents as requiring HIV testing if they reported >1 of these five criteria had 74% sensitivity and 80% specificity for HIV, with the algorithm correctly predicting the HIV status of 79% of participants. In low-HIV-prevalence settings (<2%), the algorithm would have a high negative predictive value (≥99.5%) and result in an estimated 60% decrease in the number of people needing to test to identify one HIV-infected individual, compared with universal testing.

**Conclusions:**

Our simple algorithm can identify which individuals are likely to be HIV infected with sufficient accuracy to provide a screening tool for use in settings not already implementing universal testing policies among this age-group, for example immigrants to low-HIV-prevalence countries.

## Introduction

Almost three decades after the start of the HIV pandemic, more than 2 million children worldwide are HIV-infected, with the majority living in Southern Africa (AIDS Epidemic Update, 2009). Coverage of diagnosis and treatment of HIV-infected children has lagged behind that of adults (Prendergast *et al.* 2007), although there has been an increased focus on scaling up paediatric HIV diagnosis and treatment programmes in recent years (AIDS Epidemic Update, 2009; WHO & UNAIDS, 2009). Paediatric HIV programmes have focused mainly on diagnosis and treatment of HIV infection among infants and younger children, and tended to exclude older children and adolescents, who have instead been primarily targeted for HIV prevention. (AIDS Epidemic Update, 2009).

The likelihood of survival to adolescence with undiagnosed maternally acquired HIV infection is now known to be higher than previously assumed (Newell *et al.* 2004); about a third of HIV-infected infants are ‘slow-progressors’ and thus HIV infection acquired during infancy is now a major cause of adolescent ill-health in countries with severe, longstanding HIV epidemics (Marston *et al.* 2005; Ferrand *et al.* 2009, 2010a,b). As a legacy of earlier assumptions that long-term survival with untreated vertical HIV was likely to be exceptional, underlying HIV has often not been considered in older children and adolescents presenting with acute ill-health (Eisenhut *et al.* 2008). This results in delayed diagnosis (Ferrand *et al.* 2007a; Judd *et al.* 2009) of a high risk of irreversible complications of HIV by the time diagnosis is finally made and treatment started as well as a risk for onward transmission (Ferrand *et al.* 2007b; Foster *et al.* 2007).

In Harare, Zimbabwe, HIV prevalence rates in pregnant women were high throughout the 1990s, peaking at about 30% in 1997 (Zimbabwe National HIV and AIDS Estimates 2007, 2007). HIV prevalence is now extremely high in adolescents (aged 10–18 years) accessing secondary and primary acute care services (46% and 17%, respectively) (Ferrand *et al.* 2010a,b; Ferrand *et al.* 2010a,b). Underlying HIV infection was already identified in all but a small minority of hospitalized adolescents, but was previously undiagnosed in 81% of HIV-infected adolescents accessing primary care services. At both health service levels, characteristic features suggesting long-standing or maternally acquired HIV infection were noted to be present in most cases.

In this study, we used data from primary health care in Harare to construct a simple screening tool to identify adolescents at high risk of undiagnosed HIV infection. This tool could be adapted for use at primary care level in settings where HIV testing and counselling is not routinely provided.

## Methods

### Study population and data collection

Patients aged 10–18 years attending two primary care clinics in Harare for any reason except antenatal care were enrolled consecutively into the study over a 6-month period. Patients were excluded if they were attending for antenatal care, were too ill to take part, had been previously enrolled in the study, or were younger than 16 years and not accompanied by a guardian.

All participants were offered an HIV test following group pre-test counselling and asked to consent for participation in the study, including an additional HIV test for study purposes, regardless of whether the participant accepted diagnostic HIV testing. A standardized questionnaire was used to record brief demographic details, clinical history and reason for clinic attendance. *Z*-scores for height- and weight- for age were calculated using British 1990 Growth Reference Curves (chosen because, unlike the 2006 WHO standards, these provide height and weight data for children over the age of 10 years) (Cole 1997; de Onis *et al.* 2007). Data were entered into an Access database using Epi-Info 2002 (CDC, Atlanta, USA), linked with HIV test results after removal of all personal identifiers, and exported into Stata 10 (StataCorp, Texas, USA) for analysis. The chosen sample size provided 80–90% power to detect ORs of around 3.0 or higher between the considered risk factors and HIV infection when the data set was split into two.

Written informed consent was obtained from all participants, and also from guardians of participants aged below 16 years. The study was approved by the Medical Research Council of Zimbabwe, the London School of Hygiene and Tropical Medicine Ethics Committee and the Biomedical Research and Training Institute Ethics Committee.

### Data analysis

A random number generator was used to divide the data set into two: a ‘train’ and ‘test’ data set, with an equal number of HIV-positive participants in each data set. The train data set was used to create and optimize the screening algorithm, which was then evaluated in the test data set.

### Algorithm criteria

The algorithm was designed for use at primary care level and thus variables that could be measured by primary health care workers were selected. Candidate variables used to construct the algorithm were defined *a priori* and were coded as binary variables. The considered criteria were defined as follows:

- Recurrent upper respiratory tract infections: more than two upper respiratory tract infections (URTI) over a period of at least 6 months
- Recurrent chest infections: more than two chest infections diagnosed in primary or secondary care over a period of at least 1 year
- Recurrent diarrhoea: more than 3 acute or chronic episodes of loose stool over a period of at least 6 months, with at least a week's diarrhoea-free period between each episode
- Recurrent skin problems: more than 3 episodes of any skin complaints occurring over a period of a year or more
- Possible sexually transmitted infection (STI): vaginal/urethral discharge or genital sores
- Oral candidiasis: white spots or plaques in the mouth
- Possible tuberculosis: cough >2 weeks and one of the following: night sweats, weight loss, fevers
- Poor Functional ability: Illness affecting ability to function in daily life in the past 3 months
- Wasting: weight-for-age *z*-score <−2
- Stunting: height-for-age *z*-score <−2
- Pubertal delay: Tanner Stage 1/2 in those aged 14 years or older

Other criteria that were considered included age, sex, marital status, educational level, history of TB, self-rated health and history of hospitalization (at least one night stay in hospital for any reason).

#### Construction and optimization of the algorithm

The odds ratio (OR) for the association of each variable with HIV infection was estimated. Variables with a *P*-value<0.1 were included in an initial multivariate model. Variables not independently statistically significant (i.e. with a *P*-value >0.05) were excluded from the multivariate model by stepwise backward logistic regression, so the final model included only those variables independently associated with HIV.

The log of the probability of being HIV-infected (*P*) was calculated for different combinations of variables V_i_ from the final multivariable model as follows: 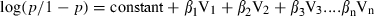
where V_i_ is the binary variable i (coded as 1 if the variable is present and as 0 if the variable is absent) and β_i_ is the log(OR) associated with variable V_i_.

The next step was to select a cut-off value of *P*, which would discriminate which individuals should be considered as being at higher risk of HIV infection and be referred for an HIV test. Using this cut-off, an algorithm which classifies individuals into one of two groups, ‘high risk for HIV’ and ‘low risk for HIV’, was devised. To choose the optimal cut-off, the sensitivity and specificity for a range of cut-offs against true HIV status was calculated. The positive predictive value (PPV), negative predictive value (NPV) and the likelihood ratio (LR) of the algorithm using different cut-offs were also calculated. To increase the sensitivity of the algorithm without compromising specificity, additional variables with very high specificity (>97%) for HIV infection were added as options to the model at the desired cut-off of *P.*

### Evaluation of the algorithm

The optimized algorithm was applied to the test data set and sensitivity, specificity, PPV and NPV and LR calculated. The PPV, NPV and number needed to HIV test to detect one HIV-positive individual were then calculated for varying HIV prevalence levels.

## Results

A total of 506 participants (97% of those eligible) were enrolled during the study period. Thirty-two attendees were excluded because of severe illness (7), no guardian available for consent (12), and consent to participate being refused (13). Two hundred and fifty-one participants were randomly assigned to the train set and 255 to the test data set. Eighty-six (17%) participants were HIV-positive, and there were 43 HIV-infected participants in each data set.

Age, sex, marital status and pubertal delay were not associated with HIV infection on univariate analysis. Table 1 shows the variables associated with HIV infection (OR ≥2.5 or *P*-value <0.05) on univariable and multivariable analysis. Orphanhood, hospitalization, recurrent skin problems, presentation with a sexually transmitted infection (STI) and poor functional ability were independently associated with increased risk of HIV infection in the multivariable analysis and were included in the algorithm. Under the multivariable logistic regression model, a cut-off of *P*=0.12 corresponded to an individual who met more than one of the five criteria in the model being considered as ‘high risk for HIV’, and hence would be offered HIV testing under the proposed algorithm.

**Table 1:** Sensitivity, specificity, crude odds ratio and adjusted odds ratio for variables associated with HIV infection

	Sensitivity (%)	Specificity (%)	Crude OR	*P*-value	Adjusted OR*	*P*-value
History of TB	9	97	3.45	0.06	4.37	0.08
Orphan	77	58	4.59	<0.001	3.93	0.002
Ever hospitalized	35	90	4.77	<0.001	4.05	0.003
≤Primary education level	63	54	2.01	0.04	1.85	0.14
Recurrent URTI	44	82	3.66	<0.001	2.41	0.06
Recurrent chest infections	37	85	3.26	0.001	1.76	0.23
Wasting	35	85	2.95	0.004	1.91	0.17
Stunting	28	86	2.39	0.03	1.64	0.34
Recurrent diarrhoea	53	74	3.20	0.001	1.66	0.25
Recurrent skin problems	51	82	4.69	<0.001	4.07	0.001
Self-rated poor health	65	79	7.16	<0.001	2.44	0.06
Poor Functional ability	51	86	6.47	<0.001	4.82	<0.001
Possible TB	12	97	3.78	0.03	2.96	0.16
Possible STI	12	96	3.29	0.05	5.35	0.015

* Adjusted for orphanhood, hospitalization, chronic skin problems, functional ability and possible STI.

### Identification of the optimum algorithm

**Figure 1:**
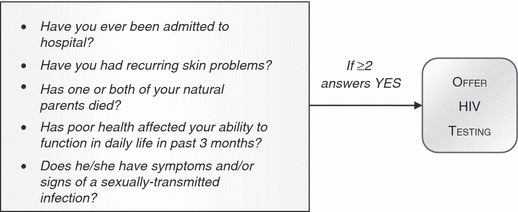
Adolescent ‘HIV suspect’ algorithm.

Using a cut-off of *P*=0.12, the sensitivity and specificity of the algorithm to predict HIV infection in the trainer data set were 77% and 81%, respectively. Lower and higher cut-offs resulted in a substantial drop in specificity and sensitivity respectively: for example, using a cut-off of *P* = 0.38 (corresponding to >2 criteria in the algorithm being met) resulted in a specificity of 95% but sensitivity dropped to 47%. Thus, other cut-offs were not considered further. High specificity variables (history of TB, history of herpes zoster and presence of oral candidiasis) were tested in various combinations as an option to the original algorithm, but resulted in no significant improvement in sensitivity of the algorithm, and thus, the original model was retained as the final algorithm (Figure 1).

### The test data set

Applying the algorithm (with a cut-off of *P =*0.12) to the test data set gave a sensitivity of 74.0% (95% CI: 64%–82%) and a specificity of 80% (95% CI:71%–87%) with the algorithm correctly classifying the HIV status of 79% of participants. As an additional check for internal validity of the model, the data set was bootstrapped; after 50 000 iterations, the sensitivity was 81% (95% CI: 73–88%) and the specificity was 77% (95% CI: 68–82%).

### Prediction of HIV Status in low-HIV-prevalence settings using the test data set

The algorithm had high estimated NPV in both low- and high-HIV-prevalence settings and would result in an estimated 60% decrease in the number of adolescents needing to test to identify one HIV-infected individual, compared with universal testing (Table 2). Using the algorithm in an adolescent clinic population with a low prevalence of HIV infection for e.g. 0.1% underlying risk of HIV infection, the algorithm would identify 377 adolescents for HIV testing for every one true positive (compared to a 1000 adolescents screened to identify one HIV-infected adolescents). However, it is also important to minimize the number of false-negative results (i.e. HIV-infected adolescents identified as being low risk): using the algorithm an individual would be falsely classified as ‘not at risk’ for every 2189 adolescents screened.

**Table 2:** Positive predictive value (PPV), Negative predictive value (NPV), number needed to test to identify 1 HIV-infected adolescent after application of algorithm (NNT+ algorithm), number needed to test to identify 1 HIV-infected adolescent misclassified by algorithm as not at HIV risk (NNT- algorithm) and reduction in NNT+ by using algorithm, at different HIV prevalence levels

HIV prevalence in acutely unwell adolescents (%)	PPV (%)	NPV (%)	NNT+ algorithm	NNT− algorithm	Reduction in NNT+ compared to universal testing (%)
0.05	0.1	100	754	4380	62
0.1	0.3	100	377	2189	62
0.5	1.3	99.8	76	437	62
1	2.6	99.5	38	218	62
2	5.1	99.1	19	108	61

## Discussion

This study shows that a simple, question-based algorithm can identify underlying HIV infection with reasonable sensitivity and specificity in African adolescent primary care attendees born into a population in which the adult HIV epidemic was at high prevalence at the time of their birth. The algorithm had a sensitivity and specificity equal to or better than other tools to identify HIV infection in children, including algorithms based on clinical signs and symptoms (Horwood *et al.* 2003; Bahwere *et al.* 2008) and even paediatrician assessment, (Horwood *et al.* 2003) and requires a very simple assessment that could be administered following minimal training. Existing paediatric algorithms to identify children with HIV infection, such as the IMCI/HIV algorithm, tend to focus on diagnosis of younger children with symptomatic HIV infection, which may not be applicable to older children, and are also less evidence based (Qazi & Muhe 2006).

We have previously established that vertically acquired HIV is likely to be responsible for most *symptomatic* HIV infections in this age-group in Zimbabwe and this may also be true for neighbouring countries (Walker *et al.* 2006; Ferrand *et al.* 2009). Maternally acquired infection is also a risk factor in older African children and adolescents who present with previously undiagnosed symptomatic HIV infection having emigrated from high- to low-HIV-prevalence countries (AIAU, NSHPC, & CHIVA, 2007; Judd *et al.* 2009). Failure to consider possible long-term survival with vertically acquired HIV can result in missed opportunities for early diagnosis, affecting survival prospects and increasing the risk of severe and irreversible long-term complications and mortality (Arpadi 2000; Buchacz *et al.* 2003). Undiagnosed HIV should, therefore, be considered in all acutely unwell adolescents from families with known risk factors for HIV (e.g. immigrants from high-HIV-prevalence settings or intravenous drug users).

Current international recommendations are that HIV testing should be routinely offered to all attendees in health facilities (WHO & UNAIDS 2007) and facility-based testing is a highly cost-effective way of identifying HIV-positive individuals even in low-HIV-prevalence settings (Paltiel *et al.* 2005; Sanders *et al.* 2005). However, in practice not all national policies are in line with the current international ones, often exclude children, and resource constraints may adversely affect implementation (WHO & UNAIDS, 2009). In settings where universal testing is not routine for this age-group, we propose a risk assessment based on the type of pre-screening algorithm presented here. As well as identifying individuals at high risk, the algorithm may serve to raise awareness among health providers of the need to consider long-term survival in acutely unwell older children and adolescents at risk of maternally acquired HIV infection.

In low-HIV-prevalence countries, most HIV testing is carried out through free-standing or sexual health services. However, many newly diagnosed HIV-infected individuals report prior consultation in primary care, implying that opportunities for earlier diagnosis are frequently missed (Sullivan *et al.* 2005; Sudarshi *et al.* 2008). Health-care workers in primary care are often reluctant to discuss HIV testing with patients, and this may be particularly true for older children and younger adolescents where considering a diagnosis of HIV will raise uncomfortable questions about the source of their infection. Use of an algorithm may then serve to prompt this process in an age-group that is not well served by alternative testing services. An additional advantage may be expansion and normalization of HIV testing in primary care, a relatively under-utilized resource for provision of HIV testing (Evans *et al.* 2009; Ma 2009).

The strengths of the study are that the data was systematically and prospectively collected, and that parallel testing with two rapid test kits was used, with discordant results resolved by retesting stored specimens with the original two tests plus an ELISA test. Hence, misclassification of HIV status will be minimal. The screening tool is simple and could be used with minimal training by clinic staff. The high prevalence of HIV infection (17%) in the study population, which was drawn from otherwise unselected adolescents attending acute care services at primary level, provided the statistical power needed to develop this type of algorithm in a relatively small sample size.

The study has several limitations, and we present this as a promising approach that can be adapted and validated according to local context. The test-train method provides an internal validation of this approach but gives no insight into external validity. The algorithm may perform differently in populations with a different mix of sexually acquired, parenterally and vertically acquired adolescent HIV infection. The positive and negative predictive values of the algorithm will vary by background HIV prevalence and prevalence of variables in the model for e.g. past TB treatment and presentation with an STI may be uncommon in adolescents who have grown up in countries where these infections are well controlled, which may lower the PPV of the algorithm. However, the sensitivity and specificity of the algorithm will remain unchanged. Data on clinical history was collected retrospectively, which could cause recall bias. However, patient-held records were checked wherever possible to verify attendances to primary and secondary care.

This is the first study to propose and evaluate a tool to identify underlying HIV infection among acutely unwell adolescents whose predominant risk factor for HIV is maternal transmission. The algorithm is simple and may serve to raise awareness of the need to consider long-term survival in acutely unwell older children and adolescents at risk of maternally acquired HIV as well as identifying individuals at high risk. We suggest, however, that performance of the algorithm should be further validated in other settings.

## References

[b1] AIAU, NSHPC & CHIVA (2007). Perinatal Transmission of HIV in England, 2002–2005.

[b2] AIDS Epidemic Update (2009).

[b3] Arpadi SM (2000). Growth failure in children with HIV infection. Journal of Acquired Immune Deficiency Syndromes.

[b4] Bahwere P, Piwoz E, Joshua MC (2008). Uptake of HIV testing and outcomes within a Community-based Therapeutic Care (CTC) programme to treat severe acute malnutrition in Malawi: a descriptive study. BMC Infectious Diseases.

[b5] Buchacz K, Rogol AD, Lindsey JC (2003). Delayed onset of pubertal development in children and adolescents with perinatally acquired HIV infection. Journal of Acquired Immune Deficiency Syndromes.

[b6] Cole TJ (1997). Growth monitoring with the British 1990 growth reference. Archives of Disease in Childhood.

[b7] de Onis M, Onyango AW, Borghi E, Siyam A, Nishida C, Siekmann J (2007). Development of a WHO growth reference for school-aged children and adolescents. Bulletin of the World Health Organization.

[b8] Eisenhut M, Sharma V, Kawsar M, Balachandran T (2008). Knowledge of their children's HIV status in HIV-positive mothers attending a genitourinary medicine clinic in the UK. HIV Medicine.

[b9] Evans HE, Mercer CH, Rait G (2009). Trends in HIV testing and recording of HIV status in the UK primary care setting: a retrospective cohort study 1995–2005. Sexually Transmitted Infections.

[b10] Ferrand RA, Luethy R, Bwakura F, Mujuru H, Miller RF, Corbett EL (2007a). HIV infection presenting in older children and adolescents: a case series from Harare, Zimbabwe. Clinical Infectious Diseases.

[b11] Ferrand RA, Miller RF, Jungmann EA (2007b). Management of HIV infection in adolescents attending inner London HIV services. International Journal of STD and AIDS.

[b12] Ferrand RA, Corbett EL, Wood R (2009). AIDS among older children and adolescents in Southern Africa: projecting the time course and magnitude of the epidemic. AIDS.

[b13] Ferrand RA, Bandason T, Musvaire P (2010a). Causes of acute hospitalization in adolescence: burden and spectrum of HIV-related morbidity in a country with an early-onset and severe HIV epidemic: a prospective survey. PLoS Medicine.

[b14] Ferrand RA, Munaiwa L, Matsekete J (2010b). Undiagnosed HIV infection among Adolescents seeking Primary Health Care in Zimbabwe. Clinical Infectious Diseases.

[b15] Foster C, Waelbrouck A, Peltier A (2007). Adolescents and HIV infection. Current Opinion in HIV and AIDS.

[b16] Horwood C, Liebeschuetz S, Blaauw D, Cassol S, Qazi S (2003). Diagnosis of paediatric HIV infection in a primary health care setting with a clinical algorithm. Bulletin of the World Health Organization.

[b17] Judd A, Ferrand RA, Jungmann E (2009). Vertically acquired HIV diagnosed in adolescence and early adulthood in the United Kingdom and Ireland: findings from national surveillance. HIV Medicine.

[b18] Ma R (2009). Time to improve HIV testing and recording of HIV diagnosis in UK primary care. Sexually Transmitted Infections.

[b19] Marston M, Zaba B, Salomon JA, Brahmbhatt H, Bagenda D (2005). Estimating the net effect of HIV on child mortality in African populations affected by generalized HIV epidemics. Journal of Acquired Immune Deficiency Syndromes.

[b20] Newell ML, Coovadia H, Cortina-Borja M, Rollins N, Gaillard P, Dabis F (2004). Mortality of infected and uninfected infants born to HIV-infected mothers in Africa: a pooled analysis. Lancet.

[b21] Paltiel AD, Weinstein MC, Kimmel AD (2005). Expanded screening for HIV in the United States--an analysis of cost-effectiveness. New England Journal of Medicine.

[b22] Prendergast A, Tudor-Williams G, Jeena P, Burchett S, Goulder P (2007). International perspectives, progress, and future challenges of paediatric HIV infection. Lancet.

[b23] Qazi SA, Muhe LM (2006). Integrating HIV management for children into the Integrated Management of Childhood Illness guidelines. Transactions of the Royal Society of Tropical Medicine and Hygiene.

[b24] Sanders GD, Bayoumi AM, Sundaram V (2005). Cost-effectiveness of screening for HIV in the era of highly active antiretroviral therapy. New England Journal of Medicine.

[b25] Sudarshi D, Pao D, Murphy G, Parry J, Dean G, Fisher M (2008). Missed opportunities for diagnosing primary HIV infection. Sexually Transmitted Infections.

[b26] Sullivan AK, Curtis H, Sabin CA, Johnson MA (2005). Newly diagnosed HIV infections: review in UK and Ireland. BMJ.

[b27] Walker AS, Mulenga V, Sinyinza F (2006). Determinants of survival without antiretroviral therapy after infancy in HIV-1-infected Zambian children in the CHAP Trial. Journal of Acquired Immune Deficiency Syndromes.

[b28] WHO & UNAIDS (2007). Guidance on Provider-Initiated Testing and Counselling in Health Facilities.

[b29] WHO & UNAIDS (2009). Towards Universal Access: Scaling up Priority HIV/AIDS Interventions in the Health Sector: Progress Report 2009.

[b30] Zimbabwe National HIV and AIDS Estimates 2007 (2007).

